# Enhanced Contactless Vital Sign Estimation from Real-Time Multimodal 3D Image Data

**DOI:** 10.3390/jimaging6110123

**Published:** 2020-11-12

**Authors:** Chen Zhang, Ingo Gebhart, Peter Kühmstedt, Maik Rosenberger, Gunther Notni

**Affiliations:** 1 Group for Quality Assurance and Industrial Image Processing, Department of Mechanical Engineering, Technische Universität Ilmenau, D-98693 Ilmenau, Germany; maik.rosenberger@tu-ilmenau.de (M.R.); Gunther.Notni@tu-ilmenau.de (G.N.); 2 Fraunhofer Institute for Applied Optics and Precision Engineering, D-07745 Jena, Germany; Ingo.Gebhart@iof.fraunhofer.de (I.G.); Peter.Kuehmstedt@iof.fraunhofer.de (P.K.)

**Keywords:** contactless vital sign estimation, multimodal imaging, multispectral imaging, structured light

## Abstract

The contactless estimation of vital signs using conventional color cameras and ambient light can be affected by motion artifacts and changes in ambient light. On both these problems, a multimodal 3D imaging system with an irritation-free controlled illumination was developed in this work. In this system, real-time 3D imaging was combined with multispectral and thermal imaging. Based on 3D image data, an efficient method was developed for the compensation of head motions, and novel approaches based on the use of 3D regions of interest were proposed for the estimation of various vital signs from multispectral and thermal video data. The developed imaging system and algorithms were demonstrated with test subjects, delivering a proof-of-concept.

## 1. Introduction

The measurement of vital signs such as heart rate, oxygen saturation (SpO_2_), respiration rate, body temperature, etc. is an important basic task in biomedical metrology. Conventional devices for such tasks mostly take contact-based measurement approaches. However, contact measurement has several disadvantages. Above all, contact with the body and skin raises the risk of skin irritation and germ contamination. Moreover, contact-based devices significantly limit the freedom of body movement of the patients, and hence, it could lead to severe discomforts. Therefore, the contactless estimation of vital signs using image sensors has continuously gained importance because of its advantages regarding hygiene and patient-friendliness.

For the estimation of heart rate and oxygen saturation, there already exist plenty of works that are based on the photoplethysmographic (PPG) measurement of skin using a color camera. In the representative works [1,2,3,4], various approaches have been proposed for heart rate estimation. The core of these approaches is the measurement of the minor temporal variation in skin color. This skin color variation occurs due to different absorption spectra of oxygenated and deoxygenated hemoglobin and should be synchronous with the heartbeat. In these approaches, the first step is the selection of a region of interest (ROI) on the face. The color values of the ROI are measured throughout the frames, and a PPG signal is obtained from them via filtering and signal fusion. Finally, the frequency of the heartbeat correlating to the skin color variation can be derived from the PPG signal.

The estimation of oxygen saturation begins with the extraction of the pulsatile (AC) and nonpulsatile (DC) components of blood flow at two different channels of the color camera. These AC and DC components can be calculated either from extracted PPG signals [5,6,7] or directly from video data [8,9,10]. Thereafter, the so-called “ratio of ratios” (RR), namely the ratio between the AC-to-DC ratios that are calculated at two different wavelengths, can be determined. Theoretically, this value is linear to the value of oxygen saturation, whereby the coefficients of this RR-to-SpO_2_ conversion are to be empirically determined.

For the estimation of respiration rate, there are two different approaches. As the low-frequency components in raw PPG signals are caused mainly by respiration, the respiration rate could be estimated from low-pass-filtered PPG signals [5,6,11,12]. A further approach is based on the use of passive thermal videos [13,14]. As exhaled air is warmer than inhaled air, breathing results in a periodic variation in the temperature in the nostrils region. Based on this effect, the respiration frequency can be estimated from the continuous measurement of nostrils temperature. Besides the respiration frequency, this thermal video-based approach can additionally provide an estimation of body temperature.

These existing approaches and methods for vital sign estimation can deliver reliable values at patients with little motion and under homogeneous and steady illumination. However, there exist two major problems in the practical implementation of these approaches and methods. The motion of patients, especially the head rotation, can disturb the selection of ROIs, which should be located at the same local position on the face in each video frame. In [6] and [10], methods for the compensation of head motion based on 2D image transformation are implemented, but these methods are limited in the case of significant head rotation, because the different 2D projections of a human face with nonplanar 3D geometry in the near-field cannot be perfectly aligned with each other using an affine or projective image transformation.

Next to the problem due to head motion, the practical lighting conditions could cause another problem, especially in the estimation of heart rate and oxygen saturation. The approaches using color cameras are based on the uncontrolled ambient light; thus, the change in the intensity of ambient light could induce artefacts, or rather, distortions in the extracted PPG signals, which can hardly be perfectly filtered out or corrected. Consequently, strong changes in ambient light intensity may lead to the true vital signs being covered by noise and artefacts in the PPG signals.

In order to solve the first problem concerning motion artefacts, 3D imaging technology offers a promising approach. Compared to 2D imaging, with perspective distortion of objects, the 3D sensor always delivers 3D surface shape data independent of perspective. With a 3D sensor, it is possible to accurately capture the head motion with all six degrees of freedom. Thus, it is feasible to realize the alignment of the head poses in different video frames using 3D rigid body transformations. On this basis, 3D ROIs could be used for vital sign estimation and tracked across all video frames, and the problem of head motion could be significantly mitigated.

A simple but effective solution to the problem of unstable light conditions is the usage of active illumination instead of ambient light. Considering the reduction in eye strain for patients, the active illumination should be irritation-free as possible. Thus, it could be conceived to light the patients using illuminators in the invisible near-infrared (NIR) spectral range. Previous investigations [15] show that heart rate estimation is fundamentally possible in the spectral range of 675–950 nm. Moreover, the estimation of oxygen saturation could also be implemented using two wavelengths in the NIR spectral range on both sides of 800 nm.

The integration of different imaging modalities into one system yields the so-called multimodal imaging. In recent years, a lot of multimodal imaging systems [16,17,18,19,20] combining at least two imaging modalities such as 3D imaging, multispectral imaging, thermal imaging, etc. were developed for different applications in biometrics, culture heritage, biomedicine, and industry. With multimodal image data, a deeper and more reliable scene interpretation can be ensured by the combined use of features from different image modalities, and complex tasks can be fulfilled with the comprehensive and informative data basis.

In this work, a multimodal imaging system was designed for the implementation of both approaches mentioned above for the enhancement of contactless vital sign estimation. In this system, a high-speed 3D sensor based on the structured light technique is aligned with various 2D cameras (color, NIR, thermal), providing a comprehensive data basis with pixel-wise registration of different image data. Based on this system, efficient novel algorithms were developed for the preprocessing of video data and the estimation of heart rate, oxygen saturation, respiration rate, and body temperature in real-time. At the end, the imaging system and algorithms were demonstrated with test subjects, and a proof-of-concept could be provided with test results.

## 2. Multimodal 3D Imaging System

**Figure 1:**
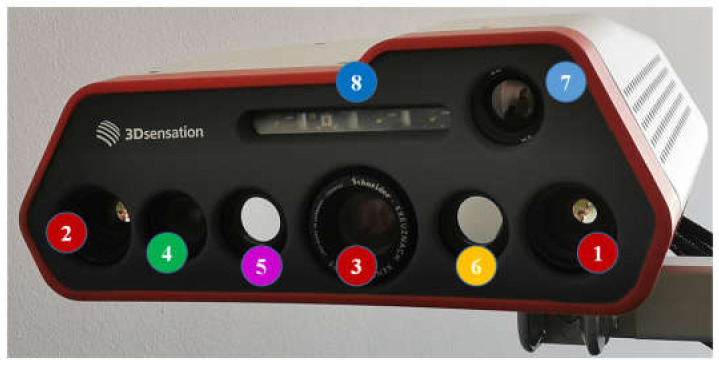
Multimodal 3D imaging system. 1/2: High-speed cameras at 850 nm, 3: High-speed GOes Before Optics (GOBO) projector at 850 nm, 4: Color camera, 5: NIR camera at 780 nm, 6: NIR camera at 940 nm, 7: Thermal camera, 8: Light emitting diode (LED) array at 780 and 940 nm.

Figure 1 shows the multimodal imaging system (manufacturer: Fraunhofer Institute for Applied Optics and Precision Engineering). It consists of a real-time 3D sensor composed of two high-speed cameras with a frame rate of 300 Hz in stereo arrangement and a high-speed GOBO (GOes Before Optics) projector [21,22]. This 3D sensor works at 850 nm and is thus irritation-free. Using a set of 10 projected light patterns, a 3D point cloud can be calculated using the stereo image correlation method in [23] with the acceleration algorithm in [24]. With the use of a graphics processing unit (GPU), a 3D frame rate up to 30 Hz can be realized, giving a real-time 3D video. Beside the 3D sensor, a color camera, two NIR cameras at 780 and 940 nm, and a thermal camera are integrated into the housing. The frame rates of these 2D cameras are up to 30 Hz, and they are hardware-based, synchronized with the 3D video stream. The entire camera system exhibits a lateral measurement field of approximately 500 mm × 400 mm at a middle working distance of 1.5 m. Furthermore, a light-emitting diode (LED) array consisting of one LED at 780 nm and three LEDs at 940 nm is used for homogeneous illumination. The beam angle (full-angle at half-maximum) of each LED is between 90° and 120°, and its power is 1 W. The total irradiation of this LED array at 1.5 m is about 1.255 µW/mm^2^ and, thus, within the eye safety limits [25]. The high-speed cameras of the stereo-vision setup and both the NIR cameras are equipped with band-pass optical filters with respective central wavelengths and a full-width at half-maximum (FWHM) of 50 nm. The GOBO projector contains a band-pass filter at 850 nm with a 50 nm FWHM in order to avoid possible spectral crosstalk between pattern projection and NIR cameras.

**Figure 2:**
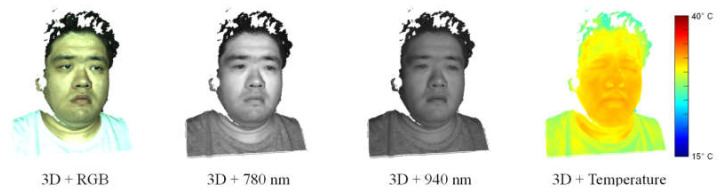
Multimodal video frame: Alignment of different 2D image data with 3D point cloud.

This multimodal 3D imaging system was calibrated using the method in [26] in order to obtain the intrinsic and extrinsic parameters of the cameras. Based on these camera parameters, 2D and 3D image data from different imaging devices can be pixel-synchronously aligned with each other and combined into multimodal video frames. The resulting multimodal 3D video has a lateral resolution of 896 × 704 pixels. Figure 2 shows a reconstructed multimodal video frame. It can be seen that the 3D point cloud of the human face was reconstructed in high quality and high resolution despite some gaps in the hair region due to its low reflectance, and the textures in different image modalities were reasonably mapped onto the 3D point cloud without any visible alignment errors. The high-quality 3D image data in the face region ensure an accurate and robust 3D face tracking, and the fine texture mapping indicates a precise superimposition of heterogeneous multispectral and thermal image data and, thus, enables an effective analysis of these multimodal image data for vital sign estimation.

## 3. Algorithms

**Figure 3:**
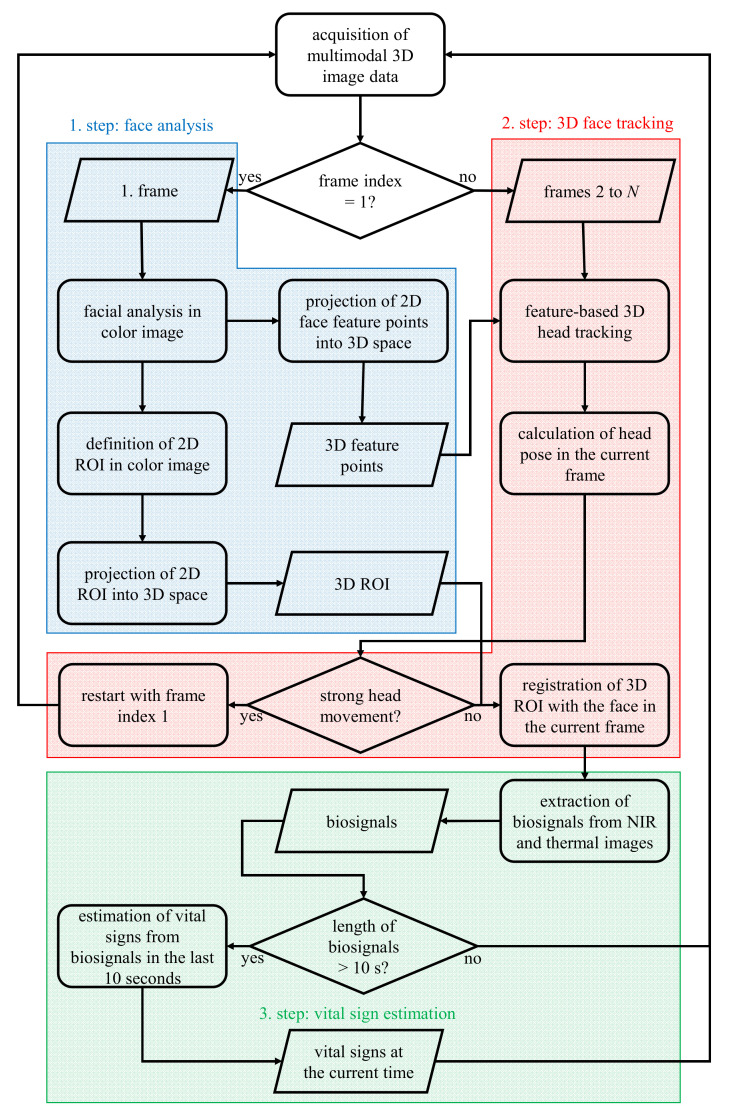
Procedure of vital sign estimation from multimodal video data.

Figure 3 outlines the procedure of vital sign estimation from multimodal video data. In general, this procedure could be divided into three steps. In the first step, front face detection is performed in the color image of the first video frame. By analyzing the detected face region, several ROIs are automatically selected for the estimation of different vital signs, and at the same time, a set of feature points are detected as preparation for the face tracking in the following video frames. In the next step, the ROI and feature points are transformed into 3D space using the camera parameters obtained from camera calibration. From the second video frame, the face is tracked based on the detected features in 3D space, so the ROI can always be located at the same places on the face in each further frame. In these ROIs, different biosignals are extracted and processed. When the length of biosignals exceeds 10 s, vital signs are continuously estimated by analyzing these biosignals in the last 10 s.

### 3.1. Face Analysis

**Figure 4:**
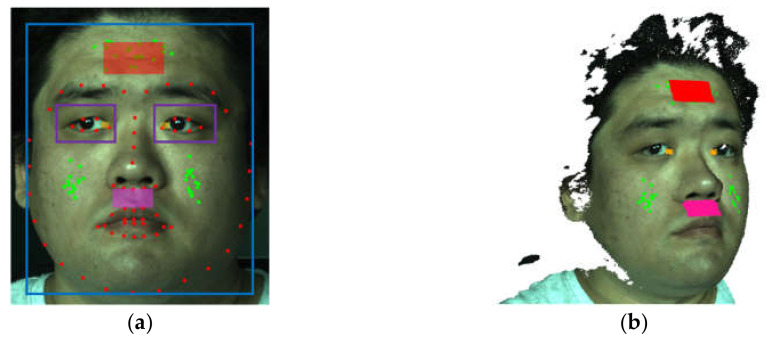
(**a**) Selection of region of interest (ROI) and feature points on the basis of face analysis in 2D color image, (**b**) transformation of ROI and feature points into 3D space.

Based on color image data collected from 30 volunteers in the laboratory, a procedure of face analysis was designed. In the color image of the first frame, the frontal face and its eye regions are detected using the Viola-Jones detector [27]. Further in the face region, the 68 facial landmarks of the OpenFace model [28] are detected based on local binary features (LBF) [29]. In the software implementation, the pre-trained cascade classifiers for face and eyes detection and the pre-trained LBF model for facial landmark detection from the library OpenCV are used. Figure 4a shows an example of detected face and eye regions (see the blue and purple windows), as well as the facial landmarks marked with red markers. On the basis of face window and facial landmarks, three ROIs are defined on the forehead (red), below the nostrils (magenta), and at the inner eye corners (orange). The highly vascularized forehead is selected for the estimation of heart rate and oxygen saturation, while the nostrils’ ROI is used for temperature-based respiration estimation. The reason for the use of inner eye corner regions for body temperature estimation is that these regions are less influenced by the ambient air flow.

For the purpose of face tracking, a set of 2D feature points, which are shown as green points in Figure 4a, are detected in the face region using the method of Shi and Tomasi [30]. In the feature point detection, the previously detected eye regions are excluded from the face windows, because these regions can be influenced by the unconscious eye motions and are thus less suitable for stable face tracking. In the end, the ROI and feature points are transformed into 3D space, so that three 3D ROIs as 3D point clouds, as well as a set of 3D feature points, are generated, as shown in Figure 4b.

### 3.2. 3D Face Tracking

From the second video frame, the Shi–Tomasi 2D feature points detected in the first video frame are first tracked in the 2D color image using the Lucas–Kanade tracker [31]. Then, the newly located feature points are also transformed into 3D space, so that corresponding 3D point pairs between the 3D face in the current frame and the face in the first frame can be created. Under the assumption that there exist very few variations in facial expression, a 3D rigid body transformation with 6 degrees of freedom (DoF) can be estimated from these point correspondences for the modeling of the current 3D head pose relating to the 3D face pose in the first frame. In order to improve the robustness of the 3D face tracking, the Kalman filter [32] was used to continuously correct the calculated face poses. Based on the 3D face tracking, strong head motions can be recognized by calculating the temporal standard deviations of the elements of the 3D head pose (translation and rotation) within a number of frames (here *N* ₌ 100) and comparing them with a set of predefined thresholds. When strong head movements are detected, the face tracking is terminated, and the next video frame is indexed with number 1 again.

**Figure 5:**
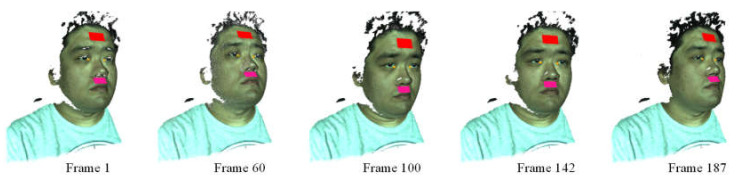
3D face tracking and registration of 3D ROIs.

In each valid video frame, the 3D ROIs generated in the first frame are registered with the face in the current video frame by transforming them into the local 3D coordinate system of the current frame using the head pose corrected by the Kalman filter. Figure 5 shows the registered 3D ROIs in different video frames. It can be seen that these ROIs are steadily and exactly located at the same local positions on the face, even if strong head rotations occur. In this way, the continuous measurement of the same skin regions despite the head rotations can be realized.

### 3.3. Vital Sign Estimation

**Figure 6:**
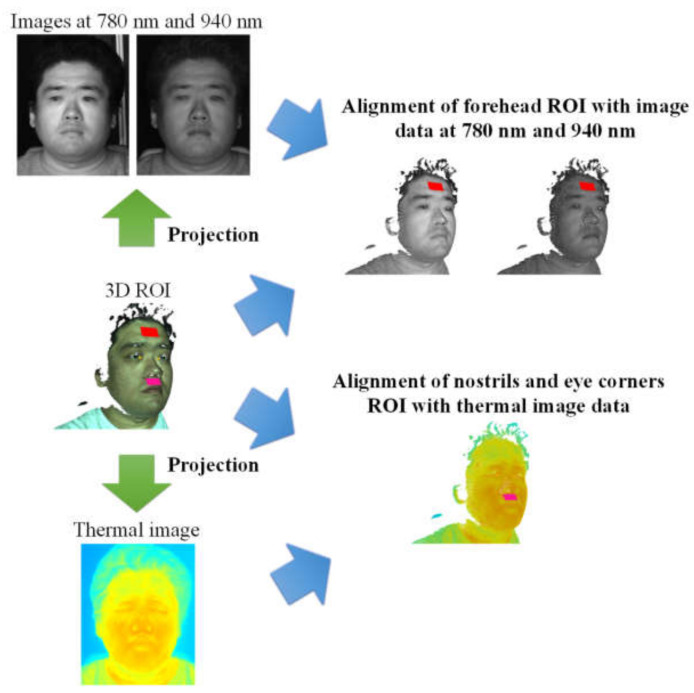
Alignment of 3D ROI with multimodal image data.

After that, the ROIs are aligned with the 3D face in the current video frame, and the 3D points of these 3D ROIs can be projected onto the images from the NIR cameras and the thermal camera in order to assign NIR and thermal grey values to these 3D ROI points. As shown in Figure 6, the forehead ROI was aligned with both the NIR images at 780 and 940 nm, while the thermal image was mapped to the nostrils and eye corners’ ROIs. For the estimation of body temperature, the mean thermal grey value of the eye corners’ ROIs was calculated and then converted to temperature values based on conversion coefficients obtained from a temperature calibration of the thermal camera.

For the estimation of respiration frequency, the mean thermal grey value of the nostrils’ ROI is calculated in each video frame. The resulting respiration signal composed of temporal mean thermal grey values is filtered using a 128-order finite impulse response (FIR) band-pass filter with a passband between 0.15 and 1 Hz. The power density spectrum of the filtered respiration signal is calculated based on discrete Fourier transformation, whereby zero-padding is performed in order to improve the resolution of frequency. Finally, the frequency of the highest peak in the power density spectrum is regarded as the estimated value of respiration rate.

The estimation of heart rate and oxygen saturation is based on the NIR cameras at 780 and 940 nm, as well as the forehead ROI. Here, there is a challenge that the signal-to-noise ratio (SNR) of the PPG measurement is comparatively low, because the skin reflectance variation is very small and could be easily corrupted by temporal noise in the NIR cameras. An approach for the improvement of the SNR of PPG signals is the Eulerian video magnification (EVM) method [10,33,34,35,36]. In this work, the EVM method is applied with some adaptations for the forehead 3D ROI to amplify the periodic variations in skin reflectance. From each 2D NIR image, a Gaussian image pyramid [37] is constructed, whereby the original image is downsampled to 4 image subscales. Then, the 3D points of the forehead ROI are projected onto each sub-image of the image pyramid, and in each image scale, the spatially filtered NIR grey values are assigned to these 3D ROI points, whereby bilinear interpolation of grey values is performed, if the coordinates of projected 2D points have sub-pixel parts. As a result, each 3D ROI point has several temporal grey value variations in different image scales. For each 3D point, its grey value variations are filtered using a 128-order FIR band-pass filter with a passband between 0.66 and 4 Hz. These temporally filtered grey value variations in different image scales are then separately amplified with different factors. For the image scale with the lowest spatial resolution, the highest amplification factor is used with an empirically chosen value of α ₌ 50, and the amplification factor linearly attenuates for higher spatial resolutions, until α ₌ 0 is reached at the highest image subscale. In the end, an output signal presenting the amplified periodic variation of skin reflectance is constructed for each 3D point by linearly combining the grey value variations that are separately filtered and amplified in each image scale.

**Figure 7:**
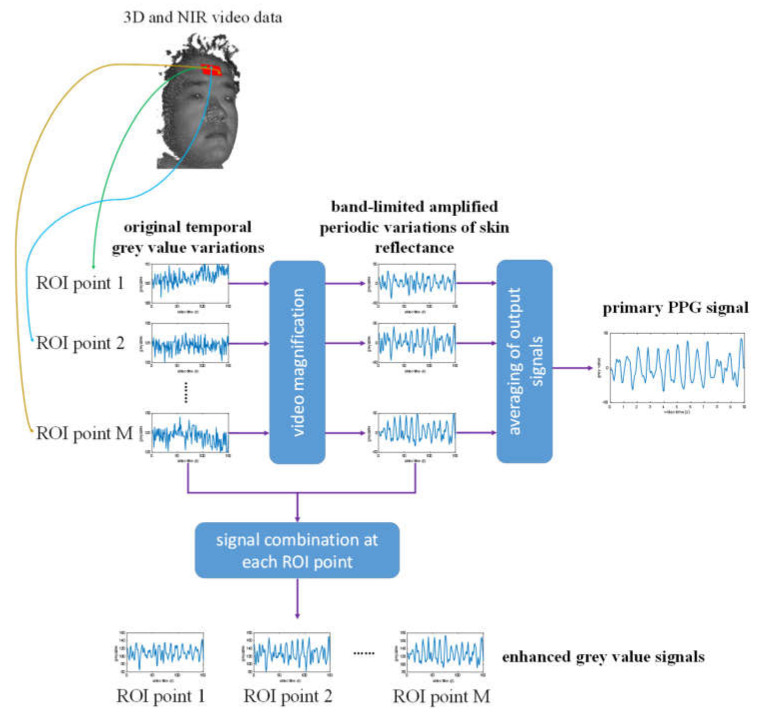
Extraction of primary photoplethysmographic (PPG) signal for heart rate estimation and enhanced NIR grey value signals for oxygen saturation estimation from 3D and NIR image data.

For the estimation of heart rate, the PPG signal is obtained from the amplified periodic variations in skin reflectance. For this, the amplified signals of all the 3D points of the forehead ROI are averaged at first, so that two primary PPG signals are generated at 780 and 940 nm, as shown in Figure 7. Thereafter, these primary PPG signals are combined by performing principal component analysis (PCA) to them, whereby the first component with the greatest variance is chosen as the final PPG signal. Similar to the estimation of respiration rate, a power density spectrum is calculated from this final PPG signal, in which the heart rate is estimated by finding the highest peak.

For the estimation of oxygen saturation, the band-limited amplified skin reflectance variations at the 3D forehead ROI points are added to their original grey value variations, resulting in enhanced NIR grey value signals, as shown in Figure 7. At each time point, the spatial standard deviation and mean value of the enhanced NIR grey values within the forehead ROI are calculated as AC and DC value. Therewith, the AC-to-DC ratio${\;R}_{\lambda }\;$can be calculated:

(1) $$R_{\lambda }\;={\;\mathrm{AC}}_{\lambda }\;/{\;\mathrm{DC}}_{\lambda }$$

In the end, the ratio of ratios RR, which should be linear to the SpO_2_ value, can be calculated by calculating the AC and DC components at both 780 and 940 nm. In order to improve the stability of oxygen saturation estimation, the RR values should be averaged over a certain time window.

(2) $$\mathrm{RR}\;={\;R}_{780}\;/{\;R}_{940}\;=\;\left({\mathrm{AC}}_{780}\;/{\;\mathrm{DC}}_{780}\right)\;/\;\left({\mathrm{AC}}_{940}\;/{\;\mathrm{DC}}_{940}\right)$$

## 4. Results

The algorithms were implemented in the C++ programming environment with the use of image processing libraries OpenCV and Point Cloud Library. The compiled program realized a real-time processing of multimodal 3D image data at a frame rate of 15 Hz. In the analysis of respiration signal, PPG signal, and signal of RR values, a 10 s sliding window was applied to estimate respiration rate, heart rate, and oxygen saturation at every video frame after the initial period. For the evaluation of the continuous estimation of vital signs, videos of test subjects with natural moderate head motions were captured in the laboratory under natural daylight, and the video duration lied between 20 and 80 s. In order to introduce variations in ground truth values of oxygen saturation, the test subjects were encouraged to hold their breath for a while during the video capturing.

For the quantitative evaluation of estimated heart rate and oxygen saturation values, a pulse oximeter “PULOX PO-200 Solo” was used as a reference device. During the acquisition of multimodal video data, the display of the pulse oximeter was captured with a webcam at a frame rate of 1 Hz. The images from the webcam were labeled with time stamps, so that the ground truth values for heart rate and oxygen saturation could be assigned to the respective frames of multimodal videos.

### 4.1. Body Temperature and Respiration Rate

**Figure 8:**
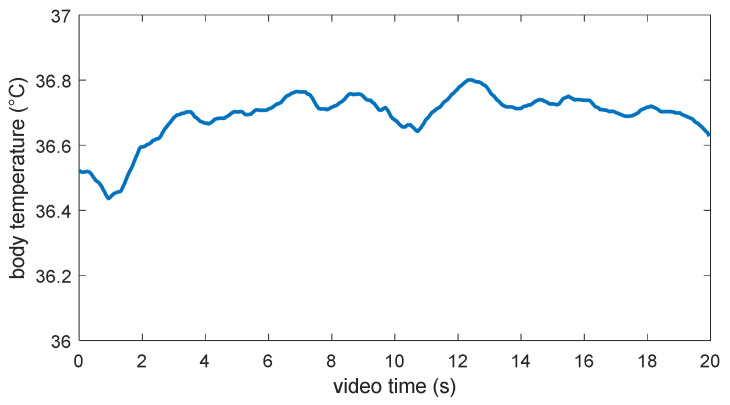
Estimation of body temperature from the eye corners’ ROIs.

Figure 8 shows the variation in estimated body temperature from the eye corners’ ROIs within 20 s. The estimated temperature value varies between 36.4 °C and 36.8 °C and appears to be reasonable with regard to the standard body temperature. The minor fluctuation may be due to the influences of air flow and the variation in the distance between eye corners and thermal cameras as a result of head motion.

**Figure 9:**
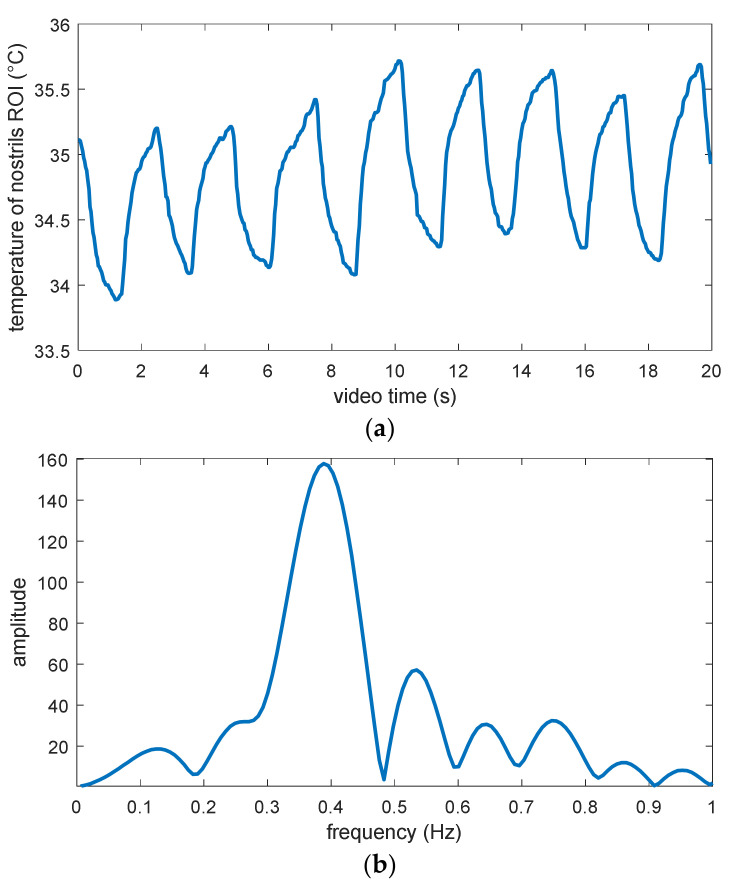
Estimation of respiration rate from the nostrils’ ROI: (**a**) Temporal temperature signal, (**b**) power density spectrum calculated from the respiration signal.

The results of respiration rate estimation are shown in Figure 9. Figure 9a shows the variation in the mean temperature of the nostrils’ ROI within 20 s. Here, an obvious periodic variation in the ROI temperature value within the range between about 34 °C and 35 °C can be seen, while the temperature contrast due to inhalation and exhalation is about 1 °C. Considering that the thermal camera exhibits a temperature resolution of about 0.05 °C, the temperature contrast in the signal can ensure a robust estimation of respiration rate against camera noise, if there is no disturbing air flow from any external source. Figure 9b shows the power density spectrum calculated from the first 10 s of this temperature signal. It presents a significant and unambiguous peak, which should correspond to the respiration rate and exhibits a frequency of about 0.39 Hz, namely 23.4 breaths per minute.

### 4.2. Heart Rate

**Figure 10:**
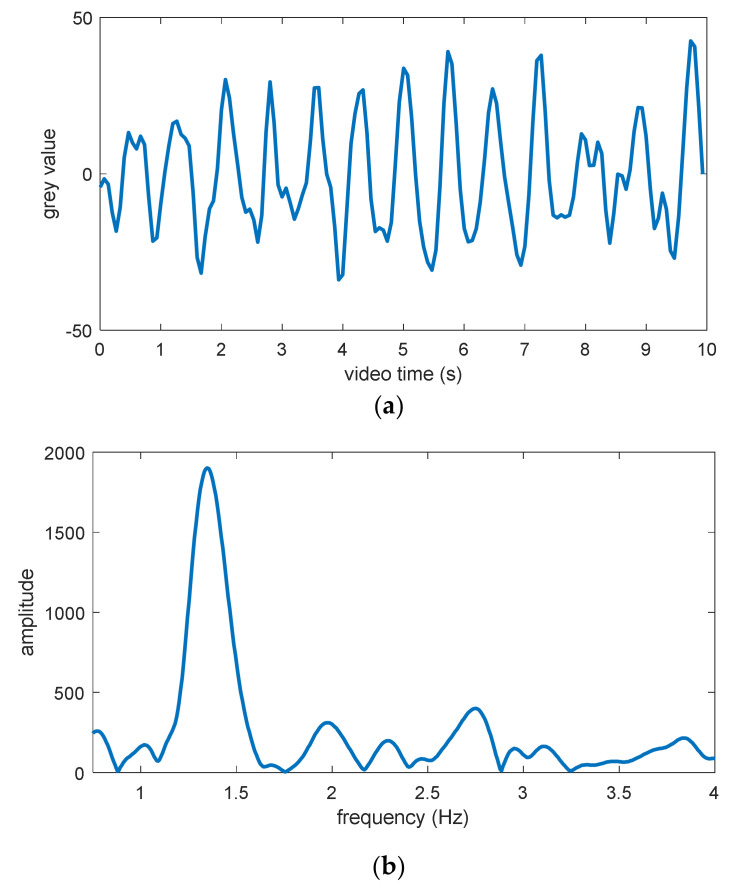
Estimation of one heart rate: (**a**) Final PPG signal, (**b**) power density spectrum calculated from the final PPG signal.

**Figure 11:**
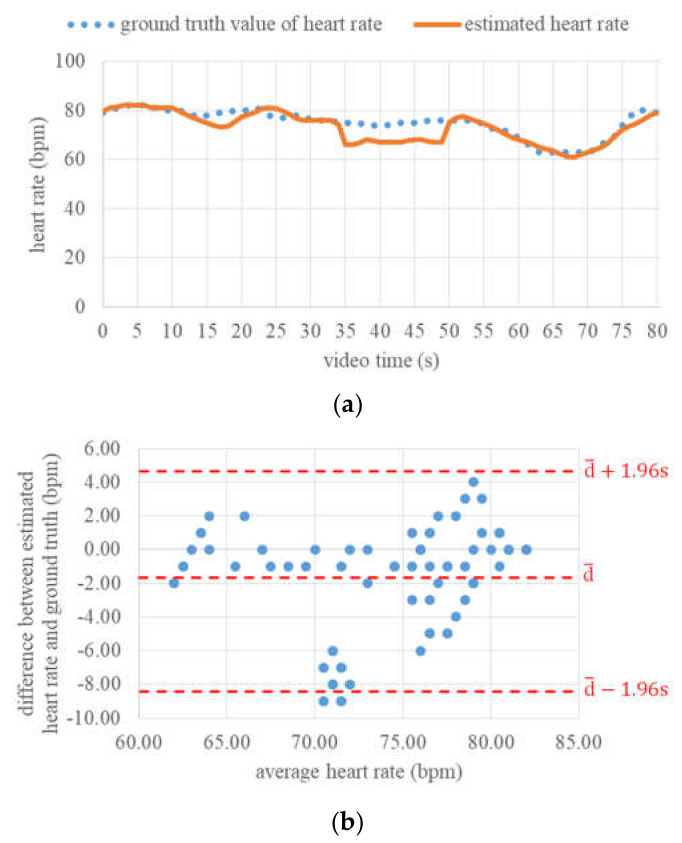
Continuous estimation of heart rate: (**a**) Ground truth values and estimated values of heart rate within 80 s, (**b**) difference between estimated heart rate and ground truth.

Figure 10 shows an example of the final PPG signal extracted from the forehead ROI and the power density spectrum calculated from this PPG signal. In this power density spectrum, an obvious peak can also be found. This peak has a frequency of approximately 1.32 Hz, corresponding to a heart rate of 79 beats per minute (bpm). This value is exactly in accordance with the ground truth value at this time point. Furthermore, in Figure 11a, the variations in estimated heart rate and ground truth heart rate within 80 s are compared with each other. It can be seen that both these variations coincide with each other well at most of the time points, except in the time range between 15 and 21 s, as well as between 33 and 49 s. It is observed that the breath of the test subject was held during both these time ranges. This action probably had certain negative influences on the heart rate estimation based on PPG measurement.

Overall, the mean absolute deviation in estimated heart rate from the pulse oximeter across all video frames was 2.56 bpm, while the maximal absolute deviation was 9 bpm. Moreover, the agreement between the contactless heart rate estimation and the pulse oximeter was assessed using the method proposed by Bland and Altman [38]. For this, the differences between estimated vital sign values and their ground truth values were determined. Then, the mean difference$\;\;\bar{d}\;$and the standard deviation of the differences s were calculated to determine the interval of agreement. For a confidence level of 95%, the limits of agreement (LoA) were$\;\bar{d}-1.96\;s$ and$\;\;\bar{d}+1.96\;s$. As shown in Figure 11b, the mean heart rate difference was −1.89 bpm, and the LoA ranged from −8.41 up to 4.63 bpm. This negative bias was due to the obviously underestimated heart rate values in both the time ranges mentioned above, but nevertheless, these values indicate that the heart rate estimation algorithm can in general deliver reasonable estimation values in the case of continuous long-time monitoring.

### 4.3. Oxygen Saturation

**Figure 12:**
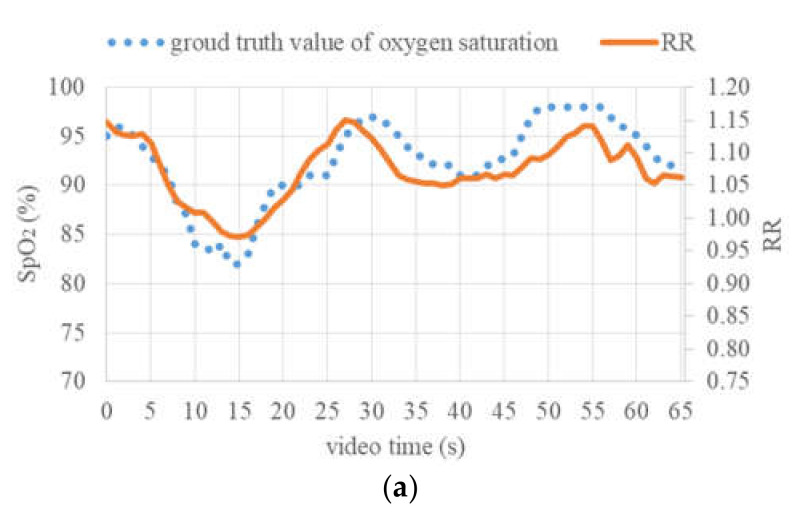

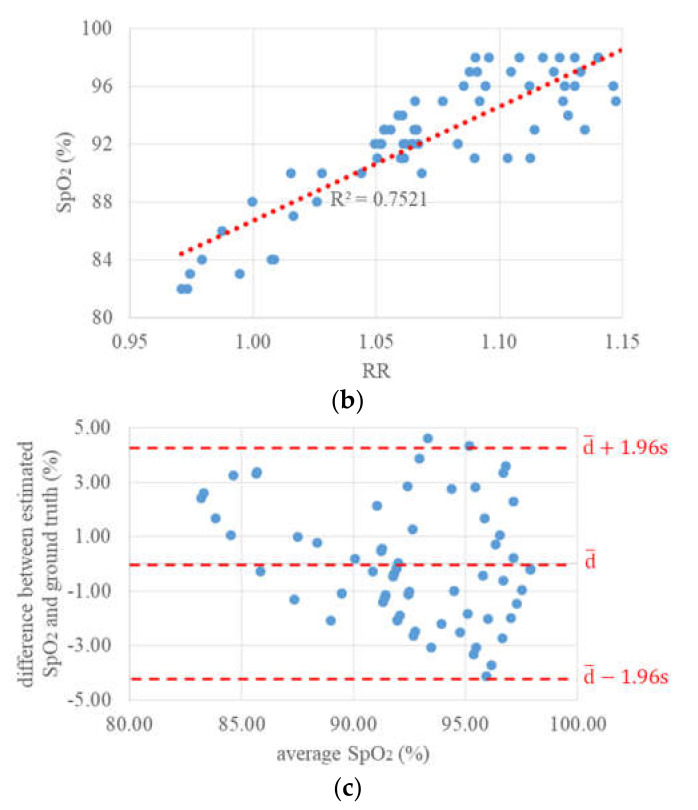
Continuous estimation of oxygen saturation: (**a**) Ground truth SpO_2_ values and ratio of ratios (RR) values within 65 s, (**b**) linear regression between RR and SpO_2_ values, (**c**) difference between estimated SpO_2_ and ground truth.

Figure 12a shows the variations in ground truth SpO_2_ values and calculated RR values within 65 s. Both these variations are qualitatively consistent with each other in the course of time, and the Pearson moment correlation coefficient between them is 0.867. In Figure 12b, the result of the linear regression between RR and SpO_2_ values is shown. In general, a linear relation can be found and the prediction of SpO_2_ values based on RR is possible, though the data appear to be somewhat noisy and the coefficient of determination R^2^ of the linear statistical model is 0.7521. Based on SpO_2_ values estimated from the RR values using this linear model, the limits of agreement were determined according to the Bland–Altman method and are shown in Figure 12c. Here, the mean difference was zero, because the same ground truth values used for the fitting of the linear RR-to-SpO_2_ conversion model were also used for the determination of LoAs, and the 95% confidence LoAs were −4.26% and 4.26%. Based on Figure 12a and the LoAs, it can be claimed that changes in the SpO_2_ values by more than 5% could be detected with high reliability.

## 5. Summary and Discussion

In this work, a real-time multimodal 3D imaging system and a hybrid approach are proposed for the enhancement of contactless estimation of heart rate, oxygen saturation, respiration rate, and body temperature. Based on real-time 3D image data, global head motions can be precisely compensated by performing 3D face tracking, while strong head movements can be detected for the assessment of measurement condition. Via the 3D sensor, multispectral and thermal image data from different 2D cameras are pixel-synchronously aligned with each other, enabling an accurate transfer of ROI among different 2D cameras and the estimation of vital signs with combined use of image data in different modalities. The demonstration shows that a real-time vital sign estimation is possible at a frame rate of 15 Hz, and the estimation of desired vital signs at subjects with moderate motions is successful in principle. What is more, further cameras could be integrated into this imaging system in order to simultaneously estimate additional vital signs such as bilirubin [39] and blood pressure [40].

In the experiments, it is noticed that the estimated oxygen saturation values are a little noisy. The reasons for this may be the imperfection in the illumination regarding spatial uniformity and remaining spectral crosstalk in the NIR cameras. On this problem, we will try to improve the LED array in order to create a more homogenous lighting and equip the NIR cameras with band-pass optical filters with narrower passbands.

Moreover, the correlation and mutual interferences between different vital signs, e.g., between heart rate and respiration, should also be systematically investigated. A joint analysis of the variations in different vital signs may offer the possibility for posterior correction of estimated values or the recognition of estimation errors.

This work presents only a demonstrative test of the system and algorithms. As preparation for the practical use, a more comprehensive evaluation in the clinical setting is planned; the estimated values will be compared with more accurate and reliable ground truth values obtained using contact-based clinical measurement devices.

## References

[1] Verkruysse W., Svaasand L.O., Nelson J.S. (2008). Remote plethysmographic imaging using ambient light. Opt. Express.

[2] De Haan G., Jeanne V. (2013). Robust pulse rate from chrominance-based rPPG. IEEE Trans. Biomed. Eng..

[3] Xu S., Sun L., Rohde G.K. (2014). Robust efficient estimation of heart rate pulse from video. Biomed. Opt. Express.

[4] Rapczynski M., Werner P., Saxen F., Al-Hamadi A. How the Region of Interest Impacts Contact Free Heart Rate Estimation Algorithms. Proceedings of the 25th IEEE International Conference on Image Processing (ICIP).

[5] Tarassenko L., Villarroel M., Guazzi A., Jorge J., Clifton D.A., Pugh C. (2014). Non-contact video-based vital sign monitoring using ambient light and auto-regressive models. Physiol. Meas..

[6] Kumar M., Veeraraghavan A., Sabharwal A. (2015). DistancePPG: Robust non-contact vital signs monitoring using a camera. Biomed. Opt. Express.

[7] Guazzi A.R., Villarroel M., Jorge J., Daly J., Frise M.C., Robbins P.A., Tarassenko L. (2015). Non-contact measurement of oxygen saturation with an RGB camera. Biomed. Opt. Express.

[8] Scully C.G., Lee J., Meyer J., Gorbach A.M., Granquist-Fraser D., Mendelson Y., Chon K.H. (2012). Physiological Parameter Monitoring from Optical Recordings with a Mobile Phone. IEEE Trans. Biomed. Eng..

[9] Bal U. (2015). Non-contact estimation of heart rate and oxygen saturation using ambient light. Biomed. Opt. Express.

[10] Rosa A., Betini R. (2020). Noncontact SpO_2_ Measurement Using Eulerian Video Magnification. IEEE Trans. Instrum. Meas..

[11] Van Gastel M., Stuijk S., De Haan G. (2016). Robust respiration detection from remote photoplethysmography. Biomed. Opt. Express.

[12] Fiedler M.-A., Rapczynski M., Al-Hamadi A. (2020). Fusion-Based Approach for Respiratory Rate Recognition from Facial Video Images. IEEE Access.

[13] Pereira C.B., Yu X., Czaplik M., Rossaint R., Blazek V., Leonhardt S. (2015). Remote monitoring of breathing dynamics using infrared thermography. Biomed. Opt. Express.

[14] Cho Y., Julier S.J., Marquardt N., Bianchi-Berthouze N. (2017). Robust tracking of respiratory rate in high-dynamic range scenes using mobile thermal imaging. Biomed. Opt. Express.

[15] Rapczynski M., Zhang C., Al-Hamadi A., Notni G. A Multi-Spectral Database for NIR Heart Rate Estimation. Proceedings of the 2018 25th IEEE International Conference on Image Processing (ICIP).

[16] Kim J., Yu S., Kim I.-J., Lee S. (2013). 3D Multi-Spectrum Sensor System with Face Recognition. Sensors.

[17] Chane C.S., Mansouri A., Marzani F., Boochs F. (2013). Integration of 3D and multispectral data for cultural heritage applications: Survey and perspectives. Image Vis. Comput..

[18] Chromy A., Klima O. (2017). A 3D Scan Model and Thermal Image Data Fusion Algorithms for 3D Thermography in Medicine. J. Healthc. Eng..

[19] Heist S., Zhang C., Reichwald K., Kühmstedt P., Notni G., Tünnermann A. (2018). 5D hyperspectral imaging: Fast and accurate measurement of surface shape and spectral characteristics using structured light. Opt. Express.

[20] Landmann M., Heist S., Dietrich P., Lutzke P., Gebhart I., Templin J., Kühmstedt P., Tünnermann A., Notni G. (2019). High-speed 3D thermography. Opt. Lasers Eng..

[21] Heist S., Lutzke P., Schmidt I., Dietrich P., Kühmstedt P., Tünnermann A., Notni G. (2016). High-speed three-dimensional shape measurement using GOBO projection. Opt. Lasers Eng..

[22] Heist S., Dietrich P., Landmann M., Kühmstedt P., Notni G., Tünnermann A. (2018). GOBO projection for 3D measurements at highest frame rates: A performance analysis. Light.

[23] Heist S., Kühmstedt P., Tünnermann A., Notni G. (2015). Theoretical considerations on aperiodic sinusoidal fringes in comparison to phase-shifted sinusoidal fringes for high-speed three-dimensional shape measurement. Appl. Opt..

[24] Dietrich P., Heist S., Landmann M., Kühmstedt P., Notni G. (2019). BICOS—An Algorithm for Fast Real-Time Correspondence Search for Statistical Pattern Projection-Based Active Stereo Sensors. Appl. Sci..

[25] International Commission on Non-Ionizing Radiation Protection (1997). Guidelines of limits of exposure to broad-band incoherent optical radiation (0.38 to 3 µm). Health Phys..

[26] Rosenberger M., Zhang C., Zhang Y., Notni G. 3D high-resolution multimodal imaging system for real-time applications. Proceedings of the SPIE 11397, Dimensional Optical Metrology and Inspection for Practical Applications IX, 27.

[27] Viola P., Jones M.J. (2004). Robust Real-Time Face Detection. Int. J. Comput. Vis..

[28] Baltrušaitis T., Robinson P., Morency L.-P. OpenFace: An open source facial behavior analysis toolkit. Proceedings of the 2016 IEEE Winter Conference on Applications of Computer Vision (WACV).

[29] Ren S., Cao X., Wei Y., Sun J. Face Alignment at 3000 FPS via Regressing Local Binary Features. Proceedings of the 2014 IEEE Conference on Computer Vision and Pattern Recognition.

[30] Shi J., Tomasi C. Good Features to Track. Proceedings of the IEEE Conference on Computer Vision and Pattern Recognition.

[31] Lucas B.D., Kanade T. An Iterative Image Registration Technique with an Application to Stereo Vision. Proceedings of the 1981 DARPA Image Understanding Workshop.

[32] Kalman R.E. (1960). A New Approach to Linear Filtering and Prediction Problems. Trans. ASME J. Basic Eng..

[33] Wu H.-Y., Rubinstein M., Shih E., Guttag J.V., Durand F., Freeman W.T. (2012). Eulerian Video Magnification for Revealing Subtle Changes in the World. ACM Trans. Graph..

[34] Bennett S.L., Goubran R., Knoefel F. Adaptive eulerian video magnification methods to extract heart rate from thermal video. Proceedings of the 2016 IEEE International Symposium on Medical Measurements and Applications (MeMeA).

[35] Dosso Y.S., Bekele A., Green J.R. Eulerian Magnification of Multi-Modal RGB-D Video for Heart Rate Estimation. Proceedings of the 2018 IEEE International Symposium on Medical Measurements and Applications (MeMeA).

[36] Ordóñez C., Cabo C., Menéndez A., Bello A. (2018). Detection of human vital signs in hazardous environments by means of video magnification. PLoS ONE.

[37] Andelson E.H., Anderson C.H., Bergen J.R., Burt P.J., Odgen J.M. (1984). Pyramid methods in image processing. RCA Eng..

[38] Bland J.M., Altman D.G. (1986). Statistical methods for assessing agreement between two methods of clinical measurement. Lancet.

[39] Munkholm S., Krøgholt T., Ebbesen F., Szecsi P.B., Kristensen S.R. (2018). The smartphone camera as a potential method for transcutaneous bilirubin measurement. PLoS ONE.

[40] Zhang G., Shan C., Kirenko I., Long X., Aarts R.M. (2017). Hybrid Optical Unobtrusive Blood Pressure Measurements. Sensors.

